# Predicting Multiple Sclerosis: Challenges and Opportunities

**DOI:** 10.3389/fneur.2021.761973

**Published:** 2022-02-08

**Authors:** Luke Hone, Gavin Giovannoni, Ruth Dobson, Benjamin Meir Jacobs

**Affiliations:** ^1^Preventive Neurology Unit, Wolfson Institute of Population Health, Barts and Queen Mary University of London, London, United Kingdom; ^2^Royal London Hospital, Barts Health NHS Trust, London, United Kingdom

**Keywords:** prediction, polygenic risk score, Multiple Sclerosis, genetics, environmental risk score

## Abstract

Determining effective means of preventing Multiple Sclerosis (MS) relies on testing preventive strategies in trial populations. However, because of the low incidence of MS, demonstrating that a preventive measure has benefit requires either very large trial populations or an enriched population with a higher disease incidence. Risk scores which incorporate genetic and environmental data could be used, in principle, to identify high-risk individuals for enrolment in preventive trials. Here we discuss the concepts of developing predictive scores for identifying individuals at high risk of MS. We discuss the empirical efforts to do so using real cohorts, and some of the challenges-both theoretical and practical-limiting this work. We argue that such scores could offer a means of risk stratification for preventive trial design, but are unlikely to ever constitute a clinically-helpful approach to predicting MS for an individual.

## Introduction

Multiple Sclerosis (MS) is a prototypical complex autoimmune disease of the central nervous system. It is the leading cause of non-traumatic neurological disability in young adults, and affects over 2 million people worldwide (1). Although the pathogenesis of MS is not completely understood, converging lines of evidence support roles for both genetic and environmental factors in determining MS susceptibility. A variety of environmental influences are associated with increased susceptibility to MS; the most consistent and replicated risk factors are smoking, childhood obesity, infectious mononucleosis, and lower serum vitamin D (2). The largest genome-wide association study (GWAS) of MS orchestrated by the International Multiple Sclerosis Genetics Consortium (IMSGC) discovered 233 genetic signals associated with MS, collectively explaining around 50% of MS heritability (3).

It may be possible to quantify an individual's susceptibility to MS based on their genetic data and exposure to certain risk factors. In principle, if it were possible to predict an individual's risk of developing MS routinely in clinical practice, this could transform all aspects of MS care, including diagnosis, treatment choices, and prognosis. Accurate and early prediction could also pave the way for trials of preventive therapies. In reality, predicting whether a given individual will develop MS may be a pipedream, as attempts to do so are constrained by several theoretical and practical challenges.

In this review we discuss previous efforts to develop MS prediction algorithms and explore the challenges facing these approaches. We present an optimistic but realistic view of how personalized prediction may enhance MS research and care over the coming decades.

## The Genetic Contribution to MS Risk

### Key Points

- MS is a complex genetic disease, with small effects of >200 loci contributing to the genetic component of risk
- Common genetic factors alone could explain up to ~20% of MS susceptibility

Heritability estimates derived from the IMSGC meta-analysis suggest that around 19.2% of MS susceptibility is attributable to the additive effects of common variants across the genome (3), of which roughly 50% could be explained in terms of genome-wide significant and suggestive effects, leaving ~50% of heritability unexplained. The strongest signal from GWAS data is for the HLA-DRB1^*^15:01 risk allele (Odds Ratio of 2.9) (3). Evidence from the IMSGC case-control exome chip study analyzing the role of rare coding variants suggests that a further ~10% of MS heritability may be explained by rare (Minor Allele Frequency < 0.05) variants (4). Neither these substantial gene discovery efforts nor smaller pedigree designs have discovered any reproducible single-gene causes of MS (5–19). Although rare monogenic forms of MS not captured in these studies cannot be excluded, these data argue for MS being a prototypical complex and polygenic disease. The genetic component to susceptibility consists in a large number of individually small effects scattered across at least two hundred genetic loci.

## Genetic Risk Scores, Environmental Risk Scores, and Prediction of MS

### Key Points

- Genetic risk scores for MS can be calculated by summing an individual's number of risk alleles at each known risk locus
- Various methods exist for deciding which risk variants to include in the genetic risk score, and how to “weight” the contribution of individual variants
- Environmental risk score's can be calculated in the same fashion if the effect of a given risk factor is known from case-control/cohort studies, and an individual's exposure to the risk factor can be quantified
- Efforts to predict MS using risk scores comprising genetic and environmental risk factors have all failed to show meaningful predictive performance on an individual level

As genotyping costs continue to fall and large biobank-scale GWAS become available for a number of common traits and diseases, it is conceivable that genotyping could become a routinely-available clinical test to help predict an individual's risk of developing a complex disease (20). If the effects of genetic variants on the risk of a disease are known through large GWAS, and an individual can be genotyped at these variants, it is straightforward to calculate the individual's genetic risk of the disease by adding together the sum of their risk alleles, each weighted by its effect: for *j* SNPs, with β_*j*_ the effect of each SNP on MS (i.e., the log odds ratio per effect allele), and *g*_*j*_ the individual's allele count at that SNP (which could be 0, 1, 2, or an imputed dosage probability), the individual's polygenic risk score over all SNPs is given by


(1) $$\begin{array}{r} PRS\;=\;\overset{j}{\underset{n=1}{\sum }}\beta_{j}g_{j} \end{array}$$

Various methods have been developed to enhance polygenic risk score prediction of complex traits (21). Although the principle is universal-to combine the effects of risk alleles across the genome using external weights derived from GWAS-these methods differ in terms of how variants are selected for inclusion in the score, and how the weights are tuned (22–25).

Large cohort and case-control studies, driven primarily by Scandinavian and North American cohorts/registry data, have consistently demonstrated that several environmental factors play a role in determining MS susceptibility (2). Such risk factors include low serum vitamin D, various aspects of EBV infection (prior infectious mononucleosis, higher anti-EBV antibody titres, EBV seropositivity in general), childhood obesity, smoking and various other putative factors such as head injury, solvent inhalation, and shift work (2). Interestingly, the effect of some of these factors appears to be potentiated by the high risk HLA allele, DRB1^*^15:01 (26–28). It is plausible that environmental risk factors for MS are modified by an individual's prior genetic risk, and if this is correct, risk models which account for gene-environment interactions are likely to outperform models which do not.

The earliest effort to predict MS using environmental and genetic data was published in 2009 (29). Since then, there have been several efforts incorporating increasingly refined genetic maps of MS susceptibility and applying this approach to novel datasets (Table 1) (29–36, 38, 39). Broadly, these studies support the view that genetic risk scores (GRS) / PRS can discriminate between cases and controls. All show moderate performance (areas under the curve, AUC, ranging from 0.52 to 0.8), but all fall short of clinically-useful diagnostic test thresholds. Efforts to demonstrate a correlation between PRS and subclinical evidence of demyelination have yielded mixed results, with the largest such cohort (~30,000 healthy controls in UK Biobank) failing to demonstrate an association (33, 40, 41) (unpublished data, https://github.com/benjacobs123456/PRS_UKB_MRI). In order to have clinical utility, scores should be able to make predictions which are useful on an individual level. The addition of disease-relevant environmental variables (such as prior smoking and prior infectious mononucleosis) has been shown to enhance the discriminative performance of these models (33).

**Table 1:** Comparison of PRS and ERS efforts in MS in literature.

**References**	**Score type**	**MS GWAS used**	**Population validated**	**Results (AUC)**
De Jager et al. (29)	PRS	16 (MHC + non-MHC)	3 populations: 2,215 cases, 1,340 cases, 143 cases	0.64–0.70
	PRS	15 (non-MHC)		0.57–0.61
	PRS + ERS	16 (MHC + non-MHC)		0.68–0.74
Jafari et al. (30)	PRS	6	Simulated 100,000 genotypes	0.64
	PRS	24		0.66
	PRS	53		0.69
Gourraud et al. (31)	PRS + Female sex	17 (MHC + non-MHC)	1,213 MS families (810 sporadic, 403 multi-case)	0.57
	PRS	17 (MHC + non-MHC)		0.55
	PRS	16 (non-MHC)		0.52
	PRS + Female sex	1 (MHC)		0.58
Disanto et al. (32)	PRS	60 (non-MHC)	70 patients, 79 HC	0.66
	PRS	110 (non-MHC)		0.69
	PRS	1 (MHC)		0.71
	PRS	61 (MHC + non-MHC)		0.77
	PRS	111 (MHC + non-MHC)		0.8
Dobson et al. (33)	PRS + ERS	1 (MHC)	78 patients, 121 unaffected siblings, 103 HC	0.77
	PRS + ERS	58 (MHC + non-MHC)		0.8
	PRS + ERS – vitamin D	1 (MHC)		0.8
	PRS + ERS – vitamin D	58 (MHC + non-MHC)		0.82
Ayati and Koyuturk (34)	PRS	8,267	975 cases	0.64–0.65
	NetPocos	243 Pocos: 3 SNPs per Pocos		0.62–0.63
Xia et al. (35)^*^	ERS	0	113 cases, 1,683 asymptomatic first degree relative	p val-0.10
	PRS	64 (MHC + non-MHC)		p val 1.5E-5
	PRS + ERS	64 (MHC + non-MHC)		p val 4.8E-6
Kulm et al. (36)	Covariates + PCA only	0	1,445 cases in UKB	0.62
	PRS + Covariates + PCA	23,309		0.73
Jacobs et al. (37)	Covariates + PCA only	0	2,276 MS cases, 486,000 controls	0.63
	PRS + Covariates + PCA	200 (non-MHC)		0.67
	PRS + Covariates + PCA	232 (MHC + non-MHC)		0.71
Barnes et al. (38)	Covariates	0	3 populations: 15 cases, 30 cases, 97 cases	0.61–0.70
	PRS + Covariates	127 (MHC + non-MHC)		0.70–0.77

* *Xia et al. (35), no AUC available, results shown as p-values for discrimination between MS cases and controls using the risk score. PRS, Polygenic Risk Score; ERS, Environmental Risk Score; PCA, Principal Components Analysis; HC, Healthy Controls*.

Although these efforts highlight the discriminative capacity of risk models *en masse*, the performance metrics are well short of what would be required for a diagnostic or predictive test. In general the methods for deriving and applying risk scores, and the reporting of the results of such analyses have been inconsistent in the literature. Few studies report absolute risk estimates within deciles of the risk scores and calibration statistics (predicted disease prevalence in each risk decile vs. observed disease prevalence). In addition there are discrepancies between studies in the methods for selecting which genetic and/or environmental factors to include in the score, the methods for generating polygenic risk scores, the statistical evaluation of the model performance, and the choice of / omission of confounding covariates such as age, sex, and genetic principal components in prediction models. Furthermore, these studies differ substantially in terms of how the data were generated, i.e., cohort characteristics, genotyping methods, and ascertainment of environmental variables. The recent development of consensus guidelines should help streamline further efforts to predict MS using risk scores (42). Given this heterogeneity in methods and reporting, it is difficult to make comparisons across published studies.

## Challenges and Opportunities for Risk Prediction Algorithms

### Key Points

- MS heritability places an upper bound on PRS performance
- Uncertainty about which variants are causal at a locus leads to inclusion of non-causal variants in PRS, which degrades performance
- Most PRS are restricted to common variants, and therefore may miss some of the susceptibility conferred by high-impact, low-frequency variants
- Modeling interactions between genetic and environmental factors may improve PRS performance over models assuming independence
- Cross-ancestry differences in LD structure and allele frequencies limit the performance of PRS in non-European ancestries
- Environmental risk factors may not be truly causal, are difficult to measure consistently, and may have varying effects over time, limiting their usefulness in risk scores
- The low prevalence of MS limits the clinical utility of all individual-level risk scores, and this is disguised by focussing on metrics like AUC, accuracy, and sensitivity/specificity rather than the positive predictive value
- Case-control definitions in biobank-scale datasets used for risk score evaluation may be imperfect
- If there are truly random processes which contribute to MS pathogenesis, these are difficult to capture with risk scores

### MS Heritability Places an Upper Bound on PRS Performance

The broad-sense heritability of MS-the proportion of phenotypic variation explained by genetic variation-places a theoretical upper limit on the performance of polygenic risk score prediction alone (43). Whilst generous estimates from twin studies estimate a broad-sense heritability of 50% (44), SNP heritability-the proportion of phenotypic variation attributable to additive effects of all typed/imputed SNPs across the genome-was estimated at 19.2% in the most recent GWAS (3). Genome-wide significant and suggestive loci only explain ~50% of this SNP heritability. These considerations emphasize the limitations of PRS generated using common, genome-wide significant markers. Even PRS which incorporate weaker effects across the genome are bounded by the $h_{SNP}^{2}$ of 19.2%. There are several explanations for missing heritability, which we discuss below, some of which could be overcome to improve MS prediction scores.

### Selecting Causal Variants for Inclusion in PRS

The classical “clumping-and-thresholding” approach to variant selection for PRS selects variants for inclusion at each independent locus (defined by an arbitrary ‘clumping' linkage disequilibrium and physical distance window), selecting the variant with the strongest statistical association with the trait (i.e., lowest *P*-value). Unfortunately, the variant with the lowest *P*-value is unlikely to be the true causal variant / one of the causal variants at the locus (45). Unless the included variant is in perfect LD (*R*^2^ = 1) with the true causal variant, the performance of the PRS will be vulnerable to the LD structure in the region, and may perform poorly even in the presence of subtly different LD (where the true causal effect will be less well-captured by the included variant). Methods incorporating local LD structure to estimate SNP effects, such as LDpred, overcome this concern to a degree and lead to appreciable improvements in prediction accuracy (23).

### Rare Variation

Rare variation may account for some of the missing heritability and thus improve PRS performance. Realistically, however, rare variants may have large effects for individuals, but they are unlikely to explain substantial phenotypic variation on a population scale. A variant with an odds ratio of 8 but a minor allele frequency (MAF) of 0.001 will only be observed, on average, once in a population of 500 people. Although this may have a substantial impact on that individual's risk of MS, it has only a limited impact on the overall performance of the score in the population.

Although heritability estimates suggest that rare (MAF < 0.05) coding variation may account for a sizeable proportion of MS heritability, the largest effort to date using the exome chip platform revealed only five associated variants within four genes outside of known MS risk loci (4). As the landscape of rare variant contributions to MS becomes clearer through large exome sequencing efforts, further performance gains may be derived from including rarer variation in PRS.

### Interactions

A simple additive PRS does not account for gene-gene or gene-environment interactions. External weights taken from GWAS assume that the effects of SNPs are constant regardless of the individual's genetic background or exposure to environmental risk factors. Various methods have been developed to account for gene-gene and gene-environment interaction in determining PRS weights. Such methods include use of conditional summary statistics, e.g., those derived from the Conditional Joint Analysis (COJO) method, which calculates effect sizes for SNPs iteratively, conditioning on each SNP in turn, starting with the strongest association (46).

Non-linear machine learning methods, such as gradient-boosted trees and random forests, can also account for high order interactions between SNPs without needing to specify these interactions *a priori*, and have been shown to afford prediction gains for complex traits in large datasets (47). It remains unclear to what extent this approach will lead to improvements in MS prediction, as widespread gene-gene interactions have not been observed outside of the MHC region in the largest sample size GWAS (3, 48). The preliminary evidence for interactions between PRS and environmental risk factors for MS suggests that incorporating GxE interaction terms into risk models may lead to further power gains (37).

### Cross-Ancestry Portability

Accurate risk estimation with PRS relies on the “true” SNP effects in the target population (i.e., the individual/s being tested) being similar to the estimated SNP effects from GWAS. Measured SNP effects in one population may differ substantially from the effect of the variant in a different ancestral population due to the different LD structures, different allele frequencies, or other factors (such as ancestry-specific gene-gene and gene-environment effects). This is a major problem for PRS derived from GWAS of individuals of European ancestry, and has been empirically demonstrated to result in poorer quality predictions for individuals of other ancestral backgrounds (49). Novel statistical methods can improve prediction in non-European populations, for instance by incorporating information from multiple ancestries (50) or prioritizing variants based on functional annotations (51). Preliminary evidence from small non-European MS cohorts suggests that the genetic architecture of MS is not identical for people with Hispanic or African ancestry (52–54). Larger GWAS of MS in non-European populations are likely to improve predictive scores for these populations.

### Environmental Risk Factors

Intuitively, including established environmental risk factors for MS should lead to improvements in prediction accuracy over genetic risk models alone. Generally, efforts to combine PRS and environmental risk factors have shown modest but appreciable improvements in discriminative performance (Table 1). Several problems limit the value of adding environmental variables to risk scores.

First, included variables may not represent truly causal risk/protective factors. Although a large number of putative environmental risk factors have been linked to MS, it remains unclear whether some of these associations are spurious, reflecting confounding and/or bias rather than causality. Mendelian randomization (MR)-an instrumental variable approach-can be used to provide further support for causality, and has added weight to the concepts that childhood obesity and low serum vitamin D are causal risk factors, whereas the evidence for smoking has been less conclusive (55–60). Clearly, inclusion of environmental risk factors which represent confounding or bias rather than causal associations may increase the noise in prediction scores and limit the utility of such scores.

Second, environmental risk factors are notoriously difficult to capture and record accurately in large cohort settings. Precise phenotype definitions, methods of testing, timing of the study (prospective vs. retrospective), and various cultural influences may lead to subtle heterogeneity in phenotype definition across cohorts, and thus the effect estimates for the effect of a risk factor in the original case-control/cohort setting may not be accurate when applied to the testing or validation cohort.

Third, unlike genetic variants which are (largely) static throughout life, environmental risk factors for MS are dynamic and time-dependent. Thus, the timing of the exposure may be critical in determining the effect on MS susceptibility. For instance, converging evidence from observational and MR designs suggests that obesity during adolescence is a risk factor for MS (59, 61, 62). Crude risk scores which consider environmental risk factors as static and binary, e.g., whether or not an individual has ever smoked or had IM prior to MS diagnosis, are a gross oversimplification and miss the time-varying effects of such exposures on the risk of MS.

Some further general concerns apply to the use of environmental risk scores, some of which also apply for genetic risk scores. These concerns include the stability and accuracy of effect estimates derived from finite sample sizes, the somewhat arbitrary choice of which variables to include, the difficulty in including relevant confounding covariates without introducing multicollinearity (e.g., controlling for socio-economic status to assess the effect of smoking status), and whether to include interaction terms in the model or consider effects as independent.

### Interpreting Performance Statistics

Most studies report the discriminative performance of PRS/hybrid risk scores, often quantified using the area under the curve (AUC) of the receiver operating characteristic (ROC) curve. The AUC can be thought of as the probability that a randomly selected case will have a higher score than a randomly selected control. Thus, the AUC is a relative measure of the risk distribution in cases vs. controls, but gives no sense of the absolute disease risk for any given individual at any point in the risk score distribution. Similarly, other metrics of overall PRS performance in a population disguise the fact that on an individual basis, prediction accuracy at an individual level often falls far short of that what would be required for a clinically-useful test. Such metrics include model fit metrics such as Nagelkerke's pseudo-R^2^ (which quantifies the proportion of variation in disease liability explained by the risk model) and the odds ratios for disease at each given PRS quantile.

For relatively rare diseases such as MS (with a population prevalence ~0.2% in the UK https://www.gov.uk/government/publications/multiple-sclerosis-prevalence-incidence-and-smoking-status/multiple-sclerosis-prevalence-incidence-and-smoking-status-data-briefing), the differences in absolute risk between deciles of the risk score are generally very small. For example, in our analysis of the >2,000 MS cases and >480,000 controls in UK Biobank, we report an impressive-sounding AUC of 0.71 for the best-performing PRS (including the MHC region). However this metric hides the fact that the difference in disease prevalence between the highest decile and lowest deciles of the PRS was only 1% (1.2% in the highest decile vs. 0.2% in the lowest decile) (37).

To illustrate this point, consider a sample population of 10,000 people with an MS prevalence of 0.5% (i.e., 50 people have MS, 9,950 people do not have MS). If the PRS distributions in cases and controls follow a standard normal, with mean = 0 in controls and mean = 3 in cases (NB this is an unrealistically large effect), a model based on PRS alone could discriminate cases from controls with an AUC of 0.98. For the purposes of a diagnostic or predictive test, a threshold needs to be established such that individuals over that threshold are considered high-risk, and those below considered low-risk.

Selecting a PRS threshold that yields sensitivity and specificity >90% identifies as high-risk all 50 people with MS (i.e., sensitivity is 100%), but also identifies 975 healthy controls as high-risk. Therefore, the positive predictive value (PPV) is only 5%, i.e., among individuals labeled as “high-risk” by the PRS cutoff, only 5% (50/975 + 50) would truly have MS.

The PPV, unlike sensitivity and specificity, depends on population prevalence (for these same parameters, the PPV would be 33% at a prevalence of 5, and 51% at a prevalence of 10%), and thus provides a more realistic means for appraising the potential clinical utility of a risk score. This illustration emphasizes why risk score prediction is more likely to be clinically useful for common traits and diseases. We have published a Shiny app to illustrate this problem (https://benjacobs.shinyapps.io/PRS_individual_predictions/).

### Case Definition for Validation of Risk Models

The evaluation of predictive models requires a large sample of cases and controls. Other than specialized disease biobanks in which MS diagnoses are rigorously checked against the McDonald criteria, case definitions for prediction studies are often derived from electronic health record (EHR) data; this is the case for most large biobanks, such as UK Biobank. Although these biobanks offer large sample sizes, especially for controls, there is a concern that EHR diagnoses may not be as accurate as McDonald-defined MS, and that some individuals may be misclassified as having MS. The high rate of MS misdiagnosis in clinical settings makes this a very real concern which could derail efforts to validate predictive scores in this setting (63).

Reassuringly, there is substantial similarity between individuals with self-reported MS and those with ICD-coded MS in UK Biobank, and the results of our analyses are unaffected by using more stringent criteria for classifying cases, e.g., restricting to individuals who have more than one source of diagnostic report (from self-report, GP records, Hospital Episode Statistics, and other sources). Although this will never achieve the accuracy of McDonald diagnosis, it is a necessary and passable simplification in our view that allows researchers to understand MS using biobank-scale data.

### Modeling Stochastic Processes

Given a generous estimate of 50% for the broad-sense heritability of MS and the individually small effects of environmental risk factors (ORs < = 3.6) (2), it is likely that a sizable proportion of MS susceptibility will remain unexplained. As discussed, there are various explanations for this explanatory gap. A particularly plausible argument is that the pathogenesis of complex diseases like MS is akin to cancer in that it involves stochastic hits which may vary from individual to individual, and are therefore difficult to measure in large cohorts. The biological underpinnings of such a process are open to speculation, but could plausibly involve events such as somatic mutations in disease-relevant tissues, aberrant breaking of immune tolerance by lymphocytes, or encountering a particular pathogen (64). A recent controversial modeling study supported this view (65). If correct, some elements of MS pathogenesis may be near impossible to quantify in a predictive model and would limit the maximum possible performance of such a model.

## Perspectives

Despite major advances in our understanding of environmental and genetic risk factors for MS, efforts to combine this information into predictive scoring systems has been disappointing. There are several theoretical reasons for this-the low population prevalence of MS, missing heritability, imprecisely-measured environmental effects, and possibly a stochastic contribution to pathogenesis which is challenging to quantify. However, there are several challenges which could be overcome. Novel approaches to polygenic risk scoring, modeling interactions between genetic and environmental factors, GWAS of non-European cohorts, and use of large biobank-scale datasets to tune and validate scores offer exciting avenues for MS prediction research. For reasons we have discussed, we are unlikely to be able to predict MS on an individual basis with an acceptable accuracy in the near future. Risk scores may, however, be useful to identify high-risk individuals to enrich populations for trials of preventive therapies, such as an EBV vaccine. In our worked example, we illustrate how a PRS could be used to identify a subset of individuals with >10x the prevalence of MS compared to the unselected population. Further work is required to ensure broad applicability of risk scores across different ancestral populations, to demonstrate the validity of such scores in prospective work, and to work with people with MS and other stakeholders to communicate the value of, and the considerable caveats surrounding, the use of predictive scoring systems in clinical settings.

## Author Contributions

BJ, LH, RD, and GG all helped conceive, write, and edit the manuscript. BJ wrote the code for the illustrations. LH wrote the first draft. All authors contributed to the article and approved the submitted version.

## Funding

This work was performed at the Preventive Neurology Unit, which is funded by Barts Charity. BJ is supported by an MRC Clinical Research Training Fellowship (Grant reference MR/V028766/1).

## Conflict of Interest

The authors declare that the research was conducted in the absence of any commercial or financial relationships that could be construed as a potential conflict of interest.

## Publisher's Note

All claims expressed in this article are solely those of the authors and do not necessarily represent those of their affiliated organizations, or those of the publisher, the editors and the reviewers. Any product that may be evaluated in this article, or claim that may be made by its manufacturer, is not guaranteed or endorsed by the publisher.
